# Oxidative Stress and Asprosin Levels in Type 2 Diabetic Patients with Good and Poor Glycemic Control

**DOI:** 10.3390/biom14091123

**Published:** 2024-09-05

**Authors:** Abdulhalim Senyigit, Sinem Durmus, Remise Gelisgen, Hafize Uzun

**Affiliations:** 1 Department of Internal Medicine, Faculty of Medicine, Istanbul Atlas University, Istanbul 34408, Türkiye; abdulhalim.senyigit@atlas.edu.tr; 2 Department of Medical Biochemistry, Faculty of Medicine, Izmir Katip Celebi University, Izmir 35620, Türkiye; durmus.sinem@gmail.com; 3 Department of Biochemistry, Cerrahpasa Faculty of Medicine, Istanbul University-Cerrahpasa, Istanbul 34320,Türkiye; remise.gelisgen@iuc.edu.tr; 4 Department of Biochemistry, Faculty of Medicine, Istanbul Atlas University, Istanbul 34408, Türkiye

**Keywords:** T2DM, glycemic control, asprosin, lipid hydroperoxides, malondialdehyde, glutathione, superoxide dismutase, total antioxidant capacity

## Abstract

**Objectives:** HbA1c is the most widely used test as an indicator of glucoregulation in patients with type 2 diabetes mellitus (T2DM). Asprosin and oxidative stress levels can be reduced with good glycemic control (GC) and thus prevented or delayed micro/macro complications in patients with T2DM. The relationship between asprosin, which is thought to affect GC, and oxidative stress parameters such as lipid hydroperoxides (LOOHs), glutathione (GSH), malondialdehyde (MDA), superoxide dismutase (Cu,Zn-SOD), and total antioxidant capacity (TAC) was evaluated in T2DM patients. **Materials and Methods:** The study was conducted prospectively in 75 healthy people admitted to the hospital for a general health check-up and 150 T2DM patients treated in the diabetes outpatient clinic. The patient’s glycemic status measurements were categorized as good glycemic control group (GGC) is defined as HbA1c < 7 and poor glycemic control (PGC) group is defined as HbA1c ≥ 7. **Results:** The study found a consistent increase in LOOH and MDA levels across the control, GGC, and PGC groups, while GSH, Cu/Zn-SOD, and TAC levels decreased in these respective groups. Additionally, asprosin levels showed a gradual rise in all groups. Positive correlations were observed between asprosin levels and various metabolic and oxidative stress markers, including BMI, WC, FBG, insulin, homeostasis model assessment for insulin resistance (HOMA-IR), DM duration, LOOH, and MDA, while negative correlations were noted with GSH, Cu/Zn-SOD, and TAC specifically in the PGC group. Furthermore, multivariate regression analysis identified HOMA-IR as the primary influencing factor on asprosin levels in PGC patients. **Conclusions:** Current glycemic dysregulation may lead to increased circulating asprosin and oxidative stress, which cause complications. Since asprosin levels may be an important hormonal factor in determining GC in T2DM, the use of this hormone may be recommended in the future to accelerate therapeutic approaches in T2DM. Early diagnosis and appropriate treatment may delay the development and progression of diabetic complications.

## 1. Introduction

Diabetes mellitus (DM) is one of the metabolic disorders that cause major health problems worldwide. Microvascular and macrovascular complications due to chronic hyperglycemia are responsible for mortality and morbidity in diabetic patients [1]. The main therapeutic goal in the management of all diabetic patients is to maintain good glycemic control (GC) to prevent macro- and microvascular complications [2]. GC is the optimal blood glucose level in a DM patient [3]. Glycemic control in type 2 DM (T2DM) patients can be assessed using three parameters: glycosylated hemoglobin (HbA1c), fasting blood glucose (FBG), and postprandial glucose (PPG). Among these, HbA1c is the gold standard for the estimation of GC [4]. The American Diabetes Association (ADA) [5] and the American Association of Clinical Endocrinologists (AACE) [6] recommend intensive glycemic control, with HbA1c targets of below 7% or 6.5%.

Asprosin is released from white adipose tissue and is found at nanomolar levels in circulation. Discovered as a new glucogenic protein adipokine, asprosin is encoded by exons 65 and 66 of the FBN1 (Fibrillin 1) gene [7]. When circulating asprosin was analyzed, it was found to increase with fasting and decrease acutely with refeeding. It was understood that asprosin has a circadian rhythm with feeding [8]. It is a glucogenic protein hormone stimulated by hunger. In rats, it was found that glucose reached the highest level in the first 30 min after administration of a dose of asprosin hormone. With the activation of compensatory hyperinsulinemia, the glucose level returned to normal at 60 min. With the increase in asprosin in plasma, hepatic glucose increased, but no effect on the glucose uptake of peripheral tissues in response to insulin was observed [9].

The balance between free radical formation and excretion is important. Extra radical formation damages the organism. If there is a certain increase in free radical formation or a certain decrease in their excretion in the cell, oxidative stress occurs [10,11]. As a result of many experimental and clinical studies, an increase in reactive oxygen species (ROS) was found in both types of diabetes [12,13,14].

Despite its importance, GC compliance was found to be low due to multiple factors. Identifying the factors’ affecting GC is crucial for initiating appropriate intervention to improve GC. No information is available on the role of asprosin in uncontrolled T2DM. The role of asprosin in uncontrolled T2DM remains unavailable. In this study, we evaluated the relationship between asprosin, which is thought to affect GC, and oxidative stress parameters such as lipid hydroperoxides (LOOHs), glutathione (GSH), malondialdehyde (MDA), superoxide dismutase (Cu, Zn-SOD), and total antioxidant capacity (TAC) among T2DM patients.

## 2. Materials and Methods

### 2.1. Subjects

This study was conducted according to the guidelines of the Declaration of Helsinki and approved by the Istanbul University-Cerrahpasa, Cerrahpasa Medical Faculty Clinical Research Ethics Committee (number of approval E-83045809-604.01-936016; Date: 6 March 2024). All subjects gave their informed consent for inclusion before they participated in the study. All subjects were of Turkish descent.

### 2.2. Study Groups

The diagnosis of diabetes mellitus was made according to the criteria of the American Diabetes Association (ADA) [15]. In this prospective study, 140 patients with T2DM who had diabetes for less than 10 years, who had not changed their treatment between two HbA1c values measured three months apart, and who had been admitted to the diabetes outpatient clinic of Medicine Hospital, Medical Faculty, Istanbul Atlas University were included.

#### 2.2.1. Control Group

A total of 75 healthy subjects who did not have any endocrine, vascular, cardiac, or inflammatory diseases were chosen for the control group (mean age: 41.547 ± 10.129 years, female/male (F/M): (25/50). They did not have diabetes or glucose intolerance, as confirmed by an oral glucose tolerance test (OGTT). Fasting plasma glucose levels < 100 mg/dL and glucose levels < 140 mg/dL at 2 h after OGTT were included in the study as healthy subjects.

#### 2.2.2. Patients Group

The patient’s glycemic status measurements were categorized as follows:

#### 2.2.3. Good Glycemic Control Group

Good glycemic control is defined as HbA1c < 7.

#### 2.2.4. Poor Glycemic Control Group

Poor glycemic control is defined as HbA1c HbA1c ≥ 7.

### 2.3. Inclusion Criteria

Individuals who had been diagnosed with T2DM without clinical complications at least one year previously, had a follow-up period of at least 1 year in the same outpatient clinic, and complied with lifestyle recommendations (diet and exercise compliance, non-smoking) were included. Individuals between 30 and 60 years old who were mentally competent were included in this study.

### 2.4. Exlusion Criteria

As aging increases oxidative stress, individuals over 60 years of age were excluded in this study. Patients who declined to participate in the study, patients diagnosed with malignancy (either cured or ongoing), patients with rheumatologic disease, pregnant women, patients with chronic kidney disease other than diabetic nephropathy, patients with chronic liver disease, patients receiving steroid therapy, patients with a known acute or chronic infection, patients with hematologic disease, and hypothyroid or hyperthyroid patients were excluded.

Patients were evaluated for retinopathy based on the results of anamnesis and examination performed by ophthalmologists at our hospital. Nephropathy was evaluated with spot urine protein, microalbumin, and creatinine results performed during routine controls in our hospital laboratory. Spot urine microalbumin/creatinine and spot urine protein/creatinine formulas were utilized. Neuropathy and macrovascular complications were evaluated by detailed anamnesis and physical examination and diagnosed according to ADA criteria [15].

### 2.5. Body Weight and Height

Body weight was measured with a margin of error of 0.1 kg using an electronic scale (Seca digital scale 0.1 precision, Hamburg, Germany), wearing only underwear. Height was measured with a Harpenden stadiometer (seca mod. 240 ce 0123, made in Germany) with an error margin of 0.1 cm. Height measurements were performed in the vertical position with bare feet, feet together and parallel, shoulder and gluteal region in contact with the wall.

### 2.6. Body Mass Index (BMI)

BMI (Weight [kg]/Height^2^ [m^2^]) was calculated using height and weight measurements.

### 2.7. Waist Circumference (WC)

Waist circumference (WC) was measured using a non-flexible tape measure at the midpoint between the lowest rib and the iliac crest while the subject was standing in underwear with the abdomen in a relaxed position and arms to the side.

### 2.8. Sample Collection and Measurements

Fasting venous blood samples were drawn between 8 and 10 am after the subjects fasted overnight (10–12 h). Blood samples were drawn from the brachial veins in the brachial fossa and placed into plain tubes and anticoagulant free tubes. The samples were centrifuged for 10 min at 4000 rpm at 4 °C. Biochemical tests were performed immediately. For the determination of other parameters, serum aliquots were frozen and stored at −80 °C immediately until they were required for further analysis.

### 2.9. Measurement of Serum Asprosin Levels

Serum asprosin levels were determined by using a commercially available human enzyme-linked immunosorbent assay (ELISA) kit according to the manufacturer’s instructions (Sunred Bioscience, Cat. No: 201-12-7193, Shanghai, China). All samples were examined twice. The minimum measurable level for serum asprosin was 0.775 ng/mL. Intra- and inter-CV for asprosin were determined to be 10% and 12%, respectively.

### 2.10. Measurement of Serum Lipid Hydroperoxides (LOOHs) Levels

The spectrophotometric determination of LOOH levels was also performed using the method of ferrous oxidation with xylenol orange version 2 (FOX2) [16]. All samples were examined twice. The coefficients of intra- and inter-assay variation were 4.4% (*n* = 20) and 5.2% (*n* = 20), respectively.

### 2.11. Measurement of Serum Malondialdehyde (MDA) Levels

Lipid peroxidation status was ascertained by the formation of MDA as an end product of fatty acid peroxidation. MDA levels were measured in plasma using the methodology of Buege and Aust [17]. All samples were examined twice. The coefficients of intra- and inter-assay variations were 3.9% (*n* = 20) and 5.7% (*n* = 20), respectively.

### 2.12. Measurement of Serum Erythrocyte Glutathione (GSH) Levels

Reduced GSH concentration was determined according to the method of Beutler et al. [18], using metaphosphoric acid for protein precipitation and 5-5′-dithiobis-2-nitrobenzoic acid for color development. The concentration of total hemoglobin was determined in hemolysate using a conventional method with Drabkin’s reagent, and a standard curve is read at 546 nm in a spectrophotometer [19].

### 2.13. Measurement of Serum Superoxide Dismutase (Cu,Zn-SOD) Activity

Cu–Zn-SOD activity was determined using the method of Sun et al. [20]. All samples were examined twice. The coefficients of intra- and inter-assay variations were 4.4% (*n* = 20) and 5.6% (*n* = 20), respectively.

### 2.14. Measurement of Serum Total Antioxidant Capacity (TAC) Levels

The TAC assay was performed according to the protocol of Benzie and Strain [21], with minor modifications as ferric-reducing antioxidant power (FRAP). All samples were examined twice. The coefficients of intra- and inter-assay variations were 4.5% (*n* = 20) and 6.0% (*n* = 20), respectively.

Biochemical parameters were determined using the enzymatic methods (Architect i2000, Abbott Park, IL, USA). Insulin levels were measured by the electrochemiluminescence immunoassay (ECLIA) method on Roche-Hitachi E170 (Roche/Hitachi MODULAR Analytics Combination Systems, Roche Diagnostics, Indianapolis, USA). HbA1c determination was based on HPLC (Variant Turbo II, Bio-Rad Laboratories, Inc., CA, USA). Homeostasis model assessment for insulin resistance (HOMA-IR) calculated by using the following formula:

HOMA-IR = Fasting glucose (mg/dL) × Fasting insulin (mU/L)/405

### 2.15. Statistical Analysis

The distribution of all analyzed parameters was assessed using the Shapiro–Wilk test. Descriptive statistics are presented as mean ± standard deviation. Group comparisons were conducted using one-way ANOVA, followed by Tukey’s post-hoc test. Correlation analysis was performed using Spearman’s rank correlation coefficient (rho). Statistical significance was defined as a *p*-value below 0.05. Multivariate regression analysis was used to determine the most effective parameter asprosin levels. All statistical analyses were conducted using the JASP software package version 0.18.3. A power analysis was conducted prior to the study to determine the appropriate sample size required to detect statistically significant differences in key parameters. Using LOOH (Lipid Hydroperoxide) as the representative parameter, the analysis indicated that to achieve 95% power at a significance level of 0.05, a minimum of 73 participants per group was necessary. Accordingly, each group in this study was designed to include at least 75 participants, ensuring adequate power for the detection of meaningful differences in the outcomes measured. According to the post-hoc power analysis, the study exhibited a power of 98.7% at an alpha level of 0.05.

## 3. Results

When the participants were evaluated in terms of antidiabetic drug use, 33.7% (n = 25) of the GGC group used only oral antidiabetics (OAD), 23.4% (n = 18) used only insulin, and 42.9% (n = 32) used both OAD and insulin, while 19% (n = 14) of the PGC group used only OAD, 14.3% (n = 11) used only insulin, and 66.7% (n = 50) used both OAD and insulin. In terms of antilipidemic agent use, 76.2% (n = 57) of the GGC group were on statins and 23.8% (n = 18) were not receiving treatment, while 85.7% (n = 64) of the PGC group were on statins and 14.3% (n = 11) were not receiving treatment. When the medications used for diabetes and hyperlipidemia were analyzed, no difference was found between the two groups.

Table 1 presents the demographic and clinical characteristics of individuals in the control and GGC and PGC groups. The BMI and WC control were progressively increasing in the GCC and PGC groups, respectively. The duration of DM was higher in the PGC group compared to the GCC group. Both systolic and diastolic blood pressure were higher in the GCC and PGC groups compared to the control group, with no significant difference between the PGC and GGC groups.

Table 2 presents the routine and investigated laboratory values of individuals in all groups. In the GGC group, compared to the control group, FBG, HbA1C, insulin, HOMA-IR, urea, GFR, total cholesterol, triglyceride, VLDL, and LDL values were found to be higher, while albumin and HDL values were found to be lower. In the PGC group, a similar trend of increase and decrease was observed in all these parameters. Additionally, HOMA-IR, creatinine, uric acid, phosphorus, urine protein, and microalbuminuria levels were also found to be higher compared to the control group. Furthermore, in the PGC group, FBG, HOMA-IR, HbA1C, urea, urine protein, microalbuminuria, and total cholesterol levels were higher compared to the GGC group.

The study revealed a progressive increase in LOOH and MDA levels among oxidative stress parameters in the control, GGC, and PGC groups, while GSH, Cu/Zn-SOD, and TAC levels exhibited a decreasing trend in the control, GGC, and PGC groups, respectively.

**Figure 1:**
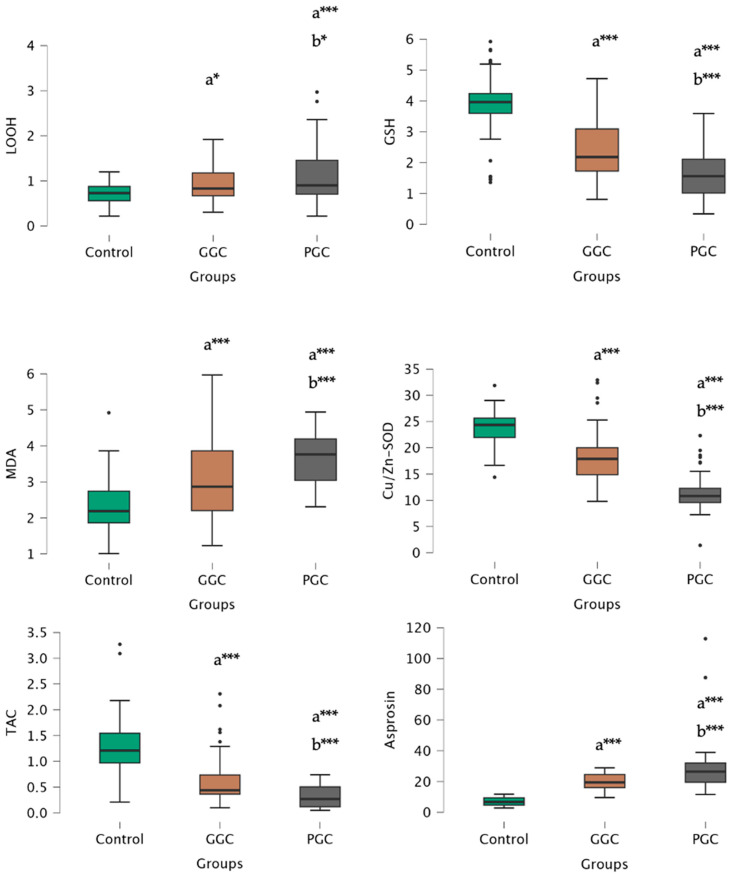
LOOH, GSH, MDA, Cu/Zn-SOD, TAC, and asprosin levels of control, GCC, and PGC groups. a: compared to control, b: compared to GCC, *: *p* < 0.05, ***: *p* < 0.001.

**Figure 2:**
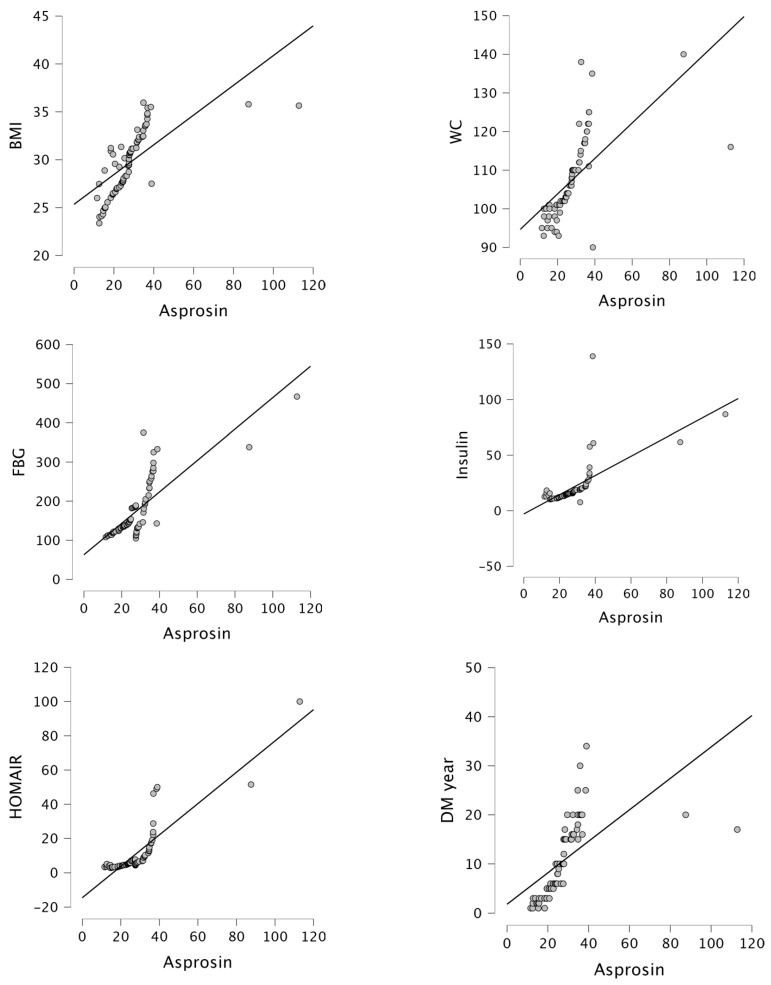
Correlation between asprosin levels and BMI (r = 0.683, *p* < 0.001), WC (r = 0.632, *p* < 0.001), FBG (r = 0.793, *p* < 0.001), insulin (r = 0.654, *p* < 0.001), HOMA-IR (r = 0.873, *p* < 0.001) and DM year (r = 0.604, *p* < 0.001) in the PGC group.

**Figure 3:**
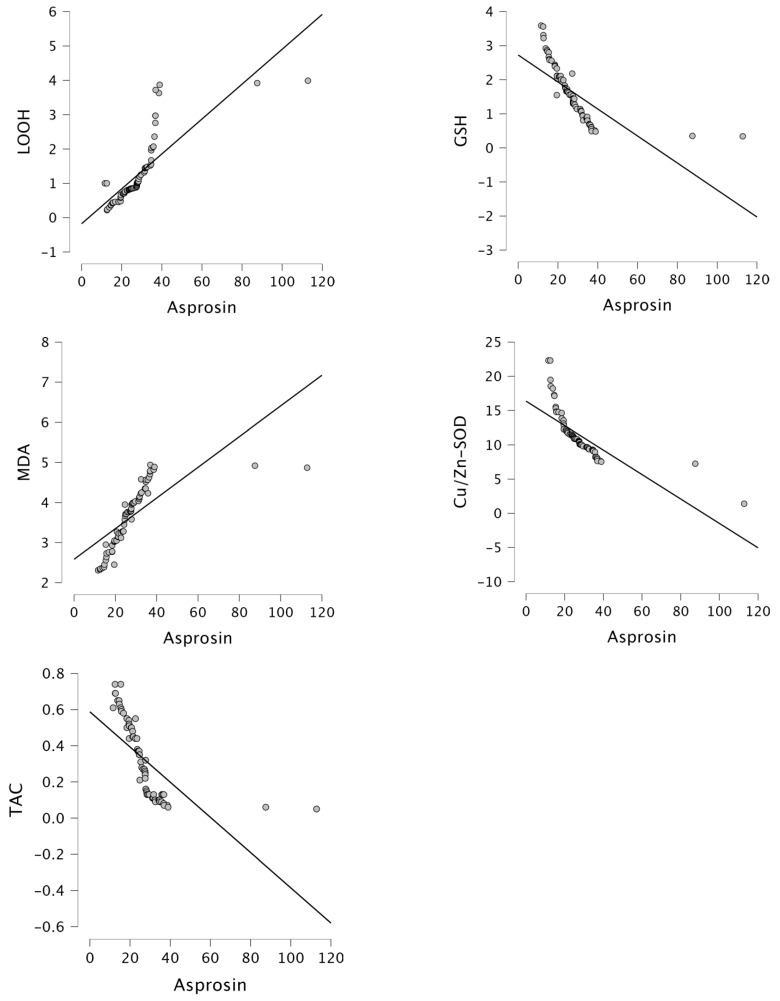
Correlation between asprosin levels and LOOH (r = 0.793, *p* < 0.001), GSH (r = −0.708, *p* < 0.001), MDA (r = 0.715, *p* < 0.001), Cu/Zn-SOD (r = −0.748, *p* < 0.001) and TAC (r = −0.658, *p* < 0.001) in the PGC group.

Moreover, asprosin levels exhibited a gradual increase in the control, GGC, and PGC groups (Figure 1). Asprosin levels positively correlated with BMI (r = 0.683, *p* < 0.001), WC (r = 0.632, *p* < 0.001), FBG (r = 0.793, *p* < 0.001), insulin (r = 0.654, *p* < 0.001), HOMA-IR (r = 0.873, *p* < 0.001), DM year (r = 0.604, *p* < 0.001), LOOH (r = 0.793, *p* < 0.001), and MDA (r = 0.715, *p* < 0.001), while negatively correlated with GSH (r = −0.708, *p* < 0.001), Cu/Zn-SOD (r = −0.748, *p* < 0.001), and TAC (r = −0.658, *p* < 0.001) in the PGC group (Figure 2 and Figure 3). Furthermore, in our study, multivariate regression analysis revealed that HOMA-IR was the factor with the greatest influence on asprosin levels in patients with PGC (Table 3).

## 4. Discussion

Our results confirmed previous reports of higher serum asprosin concentrations in diabetic patients [22,23,24,25]. Similar to previous studies, asprosin positively correlated with BMI and WC in T2DM [25,26,27,28]. In the current study, asprosin levels positively correlated with glucose, insulin, HOMA-IR, MDA, and LOOH, while negatively correlated with GSH, Cu/Zn-SOD, and TAC in the PGC group. This finding supports that asprosin is a molecule that plays an important role in IR. Asprosin could also be a sign of IR in T2DM. Our results reveal that oxidative stress and asprosin levels are increased in uncontrolled diabetes.

In the current study, in the PGC group, diabetes duration, urea, urine protein, microalbuminuria, and total cholesterol levels were higher compared to the GGC group. The relationship between disease duration and glycemic control in T2DM has been examined by many researchers, and an increase in the frequency of complications with an increase in disease duration has been observed in almost all of the studies [29,30,31]. Ghouse et al. [32] report that significant risk exists in both the higher and lower tails of glycaemia in elderly patients with longer diabetes duration. As the duration of diabetes increases, GC worsens, which has also been shown in our study. The parameters such as diabetes duration, urea, urine protein, microalbuminuria, and total cholesterol were most closely related to PGC. However, further studies are warranted to confirm the present findings and to establish the potential causal relationship between these parameters and GC.

The mechanism underlying the onset of diabetes is very complex. Because oxidative stress can cause hyperglycemia, and hyperglycemia can cause oxidative stress. In a previous study, oxidative stress contributes to IR, impairing the ability of insulin to facilitate glucose uptake into cells [33,34,35]. In the current study, LOOH and MDA levels increased in the PGC groups, while GSH, Cu/Zn-SOD, and TAC levels showed a decreasing trend in the PGC groups. Asprosin levels positively correlated with MDA and LOOH, while negatively correlated with GSH, Cu/Zn-SOD, and TAC in the PGC group. The etiology of oxidative stress in diabetes is caused by many different mechanisms. The balance between free radical formation and excretion is important. Excess radical formation damages the organism. Oxidative stress occurs if there is a certain increase in free radical formation or a certain decrease in their excretion in the cell. This study revealed that better GC in T2DM patients without clinical complications was associated with reduction in possible sources of oxidative stress, decreased shifts in redox balances, increased GSH, and defense enzymes such as SOD. The results of the present study suggest that redox homeostasis and oxidative stress are critical both in the control and development of T2DM.

Asprosin levels were found to be high in T2DM cases [22,23,24,25,28]. In the current study, asprosin levels were increased in both GGC and PGC groups compared to the control group. Asprosin positively correlated with BMI and WC in T2DM. In studies conducted in metabolic disease groups such as T2DM and polycystic ovary syndrome (PCOS), a positive correlation was found between asprosin levels and BMI [25,28,36,37]. Asprosin levels positively correlated with glucose, insulin, and HOMA-IR in the PGC group. This finding supports that asprosin could be a molecule that plays an important role in IR.

The study also found a positive correlation between asprosin and glucose, insulin, and HOMA-IR levels in both the GGC and PGC groups. The correlation coefficient in the PGC group (0.800) was higher than that in the GGC group (0.617). Zhang et al. [28] found that asprosin, which is involved in fasting glucose homeostasis, is an independent risk factor for T2DM in a T2DM patient group. A positive correlation was also found between asprosin and FBG in the T2DM group. In another study in which impaired glucose tolerance and T2DM patient groups were compared with the normoglycemic group, it was reported that asprosin level was associated with impaired glucose tolerance and T2DM. In the same study, a positive correlation was found between asprosin level and FBG [25]. In a study including T2DM and polycystic ovary syndrome (PCOS) patient groups and a healthy control group, it was found that asprosin level was positively correlated with FBG and HOMA-IR both in the whole group and in the T2DM and PCOS groups [37]. Our study revealed that worse GC and IR were associated with increased oxidative stress and asprosin levels in T2DM patients without clinical complications.

The main components of the metabolic syndrome (MetS) are dysregulated glucose homeostasis, elevated arterial blood pressure, dyslipidemia, abdominal obesity, and/or IR. Hong et al. [38] showed in a 293-subject study conducted in 2021 that serum asprosin levels tended to increase in subjects with MetS compared to controls. Furthermore, our study revealed that in patients with PGC, the factor most significantly influencing asprosin levels in multivariate regression analysis was HOMA-IR. Multivariate regression analysis revealed that HOMA-IR was the factor with the greatest influence on asprosin levels in patients with PGC. We believe that asprosin, secreted from white adipose tissue, plays a crucial role in IR. Asprosin is a novel metabolic-regulated adipokine that is considerably associated with IR. It has been suggested that asprosin antibodies may be a treatment option to prevent appetite, especially in MetS and T2DM [9]. Mishra et al. [39] demonstrate that anti-asprosin mAbs are dual-effect pharmacologic therapy that targets two key pillars of MetS—overnutrition and hyperglycemia. The current study suggests that beyond classical treatments for diabetes, the development of antioxidants or protective agents and anti-asprosin mAbs to maintain redox hemostasis may offer interesting therapeutic options for diabetes aimed at improving the regulation of blood glucose and thus the evolution of diabetic patients.

### Limitations of Study

Since asprosin can be released by adipose tissue, the asprosin levels might have been affected by how much fat tissue a patient had. Further studies can be performed to assess the effect of fat content on asprocin levels. Secondly, the c-peptide levels were not available on all patients that were included in the study, and thus we could not include those for analysis. C-peptide might have been a more accurate analysis for insulin levels since c-peptide demonstrates only internal insulin. Some of our patients were using insulin, which could affect the measured insulin levels.

An increase in asprosin levels in uncontrolled T2DM patients can cause a decrease in metabolic activity, can prevent sufficient energy production in patients, and therefore can result in increased oxidative stress in patients. Our study also revealed that asprosin may contribute to the development of diabetes by regulating glucose homeostasis, insulin secretion, and insulin resistance. Serum asprosin levels were lower in patients with GGC, and it was concluded that asprosin could be a good indicator of metabolic control. Antioxidant defenses and low asprosin levels may protect cellular homeostasis against oxidative damage in uncontrolled T2DM. Due to the significant role of asprosin levels in the regulation of T2DM blood glucose, the use of anti-asprosin monoclonal antibodies (mAbs) is suggested to expedite therapeutic approaches for T2DM. However, the lack of sufficient data on asprosin levels in uncontrolled T2DM in the literature necessitates further studies examining larger patient cohorts to support our findings.

## Figures and Tables

**Table 1:** Demographic and Clinical Characteristics.

	Control (n = 75)	GGC (n = 75)	PGC (n = 75)
**Age (years)**	54.320 ± 12.681	54.427 ± 8.360	58.040 ± 10.120
**Body mass index (BMI) (kg/m^2^)**	23.575 ± 1.349	28.201 ± 2.116 a ***	29.630 ± 3.250 a ***, b ***
**Waist Circumference (cm)**	94.773 ± 13.943	101.680 ± 12.672 a **	107.320 ± 10.390 a ***, b *
**Male/Female (n/n)**	50/25	38/37	32/43
**Diabetes duration (years)**	0	5.220 ± 3.947	10.613 ± 7.568 b ***
**Systolic blood pressure (mmHg)**	116.533 ± 10.558	132.333 ± 13.006 a ***	137.000 ± 14.704 a ***
**Diastolic blood pressure (mmHg)**	72.933 ± 6.733	78.933 ± 9.090 a ***	81.467 ± 9.255 a ***

Data are given as Mean ± Std. Deviation. a: vs. control group, b: vs. GCC group, *: *p* < 0.05, **: *p* < 0.01, ***: *p* < 0.001.

**Table 2:** Laboratory characteristics of subjects.

	Control (n = 75)	GGC (n = 75)	PGC (n = 75)
**FBG (mg/dL)**	90.708 ± 7.085	145.665 ± 55.067 a ***	173.653 ± 72.350 a ***, b **
**HbA1C (%)**	5.581 ± 0.324	6.371 ± 0.421 a ***	8.913± 1.533 a ***, b ***
**Insulin (µIU/mL)**	6.714 ± 1.046	14.977 ± 9.062 a ***	20.941 ± 18.900 a ***, b **
**HOMA-IR**	1.505 ± 0.263	5.485 ± 4.328 a *	10.702 ± 15.002 a ***, b ***
**Creatinine (mg/dL)**	0.730 ± 0.182	0.824 ± 0.258	0.922 ± 0.419 a ***
**Urea (mg/dL)**	12.216 ± 3.861	15.381 ± 5.269 a **	18.398 ± 10.569 a ***, b *
**Uric Acid (mg/dL)**	4.958 ± 1.404	5.233 ± 1.452	5.752 ± 1.900 a **
**Total protein (g/dL)**	7.037 ± 0.295	6.912 ± 2.494	6.615 ± 0.556
**Albumin (g/dL)**	4.392 ± 0.234	4.040 ± 0.578 a ***	3.848 ± 0.590 a ***, b *
**Calcium (mg/dL)**	9.114 ± 0.320	9.205 ± 0.377	9.132 ± 0.663
**Phosphorous (mg/dL)**	3.308 ± 0.481	3.542 ± 0.626	3.681 ± 0.731 a ***
**Urine protein (mg/g)**	9.309 ± 4.946	16.200 ± 36.304	39.467 ± 67.367 a ***, b **
**Microalbuminuria**	7.791 ± 17.198	13.157 ± 28.390	146.416 ± 284.968 a ***, b ***
**Urinary creatinine (IU/g creatinine)**	104.093 ± 35.538	105.426 ± 70.805	101.227 ± 75.408
**GFR (mL/min/1.73m^2^)**	105.705 ± 13.722	123.516 ± 43.236 a **	102.171 ± 45.759 b **
**Total cholesterol (mg/dL)**	175.000 ± 12.965	200.260 ± 29.690 a ***	214.691 ± 32.868 a ***, b **
**Triglyceride (mg/dL)**	108.097 ± 49.473	131.260 ± 41.733 a *	151.525 ± 79.088 a ***
**VLDL (mg/dL)**	21.619 ± 9.895	26.252 ± 8.347 a *	30.305 ± 15.818 a ***
**HDL (mg/dL)**	48.952 ± 8.631	36.645 ± 7.493 a ***	38.947 ± 7.022 a ***
**LDL (mg/dL)**	104.429 ± 16.489	137.363 ± 30.130 a ***	145.439 ± 30.745 a ***
**LOOH (nmol/mL)**	0.717 ± 0.216	0.952 ± 0.405 a *	1.225 ± 0.917 a ***, b *
**GSH (mg/dL)**	3.802 ± 1.019	2.449 ± 0.947 a ***	1.633 ± 0.801 a ***, b ***
**MDA (nmol/mL)**	2.372 ± 0.786	3.054 ± 1.029 a ***	3.639 ± 0.765 a ***, b ***
**Cu/Zn-SOD (U/L)**	23.831 ± 3.227	18.173 ± 4.655 a ***	11.446 ± 3.413 a ***, b ***
**TAC (µg ascorbic acid equivalent/mL)**	1.281 ± 0.529	0.625 ± 0.442 a ***	0.319 ± 0.212 a ***, b ***
**Asprosin (ng/mL)**	7.067 ± 2.712	20.194 ± 5.297 a ***	27.583 ± 14.302 a ***, b ***

FBG, Fasting blood glucose; HOMA-IR, Homeostatic model assessment for insulin resistance; Data are given as Mean ± Std. Deviation. a: vs. control group, b: vs. GCC group, *: *p* < 0.05, **: *p* < 0.01, ***: *p* < 0.001. FBG: Fasting blood glucose.

**Table 3:** Regression analysis results.

Parameters	Unstandardized	Standard Error	Standardized	t	*p*
BMI	0.292	0.434	0.066	0.671	0.504
FBG	−0.019	0.022	−0.098	−0.885	0.380
Insulin	−0.297	0.088	−0.393	−3.360	0.001
LOOH	−5.780	3.179	−0.371	−1.818	0.074
HOMAIR	1.257	0.163	1.318	7.726	<0.001
GSH	−2.534	4.280	−0.142	−0.592	0.556
MDA	−0.170	4.434	−0.009	−0.038	0.969
Cu/Zn-SOD	−0.170	0.714	−0.041	−0.238	0.813
TAC	−20.740	11.380	−0.307	−1.823	0.073

## Data Availability

The data underlying this article are available in the article. If needed, please contact the corresponding author. The email address is hafize.uzun@atlas.edu.tr.
